# Validation Study of the Accuracy of Echocardiographic Measurements of Systemic Blood Flow Volume in Newborn Infants

**DOI:** 10.1016/j.echo.2013.08.019

**Published:** 2013-12

**Authors:** Benjamim Ficial, Anna E. Finnemore, David J. Cox, Kathryn M. Broadhouse, Anthony N. Price, Giuliana Durighel, Georgia Ekitzidou, Joseph V. Hajnal, A. David Edwards, Alan M. Groves

**Affiliations:** a Imperial College and MRC Clinical Sciences Centre, London, United Kingdom; b Patologia e Terapia Intensiva Neonatale, Università degli Studi di Verona, Verona, Italy; c Department of Perinatal Imaging and Health, King's College London, London, United Kingdom

**Keywords:** Echocardiography, Phase-contrast MRI, Preterm infants, LOA, Limits of agreement, LVO, Left ventricular output, MRI, Magnetic resonance imaging, PC, Phase-contrast, RI, Repeatability index, SVC, Superior vena caval, VTI, Velocity-time integral

## Abstract

**Background:**

The echocardiographic assessment of circulatory function in sick newborn infants has the potential to improve patient care. However, measurements are prone to error and have not been sufficiently validated. Phase-contrast magnetic resonance imaging (MRI) provides highly validated measures of blood flow and has recently been applied to the newborn population. The aim of this study was to validate measures of left ventricular output and superior vena caval flow volume in newborn infants.

**Methods:**

Echocardiographic and MRI assessments were performed within 1 working day of each other in a cohort of newborn infants.

**Results:**

Examinations were performed in 49 infants with a median corrected gestational age at scan of 34.43 weeks (range, 27.43–40 weeks) and a median weight at scan of 1,880 g (range, 660–3,760 g). Echocardiographic assessment of left ventricular output showed a strong correlation with MRI assessment (*R*^2^ = 0.83; mean bias, −9.6 mL/kg/min; limits of agreement, −79.6 to +60.0 mL/kg/min; repeatability index, 28.2%). Echocardiographic assessment of superior vena caval flow showed a poor correlation with MRI assessment (*R*^2^ = 0.22; mean bias, −13.7 mL/kg/min; limits of agreement, −89.1 to +61.7 mL/kg/min; repeatability index, 68.0%). Calculating superior vena caval flow volume from an axial area measurement and applying a 50% reduction to stroke distance to compensate for overestimation gave a slightly improved correlation with MRI (*R*^2^ = 0.29; mean bias, 2.6 mL/kg/min; limits of agreement, −53.4 to +58.6 mL/kg/min; repeatability index, 54.5%).

**Conclusions:**

Echocardiographic assessment of left ventricular output appears relatively robust in newborn infant. Echocardiographic assessment of superior vena caval flow is of limited accuracy in this population, casting doubt on the utility of the measurement for diagnostic decision making.

Echocardiographic assessments of circulatory function show significant potential to enhance the circulatory management of infants admitted to neonatal intensive care.^1^, ^2^, ^3^, ^4^ A consensus statement on targeted neonatal echocardiography was recently released by the American Society of Echocardiography to guide practice in this area.^5^ The statement also highlights how prone to error quantitative measures are in newborns and reinforces the need for measurements of blood flow (left ventricular output [LVO] and superior vena caval [SVC] flow volume) to be standardized and validated.^5^

Phase-contrast (PC) magnetic resonance imaging (MRI) is a highly validated technique in adults^6^ and children^7^ and has recently been successfully applied to preterm infants.^8^ Assessments can be performed during natural sleep without the need for anesthesia or sedation,^8^ and scans can be performed on magnetic resonance systems located within neonatal intensive care units, allowing maintenance of cardiorespiratory and thermal stability even in the most preterm infants.^9^ PC MRI can assess flow volume in any large vessel.^10^ Critically, PC MRI is at least twice as repeatable as echocardiography in the preterm population,^8^ and with improvements in imaging resolution, measures of flow volume can now be quantified to within ±11% to 13%.^11^

The aim of this study was to use PC MRI to validate measures of cardiac output and systemic blood flow in newborn preterm and term infants.

## Methods

All echocardiographic and PC MRI scans were carried out for research purposes only, with research ethics committee approval and signed parental consent. Infants were recruited from stable infants admitted to the neonatal intensive care unit or postnatal ward at Queen Charlotte's and Chelsea Hospital, London, at any time during admission.

### Echocardiographic Measures

Echocardiographic images were acquired using a Vivid 7 ultrasound machine (GE Healthcare, Milwaukee, WI) with a 10-Mhz sector probe. Before the study of this cohort, image acquisition settings were optimized for preterm infants. All examinations were either performed by or directly supervised by a neonatologist with >10 years' experience in functional echocardiography (A.M.G.). All echocardiographic examinations were performed with infants asleep or quietly awake. No sedation was used. All subjects were screened for congenital heart disease, including the exclusion of bilateral superior venae cavae. In all cases, echocardiography was performed within 1 working day of MRI. Images were stored digitally and analyzed offline to minimize the duration of echocardiography. An additional cohort of infants was subsequently examined by echocardiography to investigate the repeatability of quantification of SVC flow volume. Examinations in these infants were all performed by a single investigator (B.F.) after standardization of approach between operators in 10 infants.

### LVO

**Figure 1:**
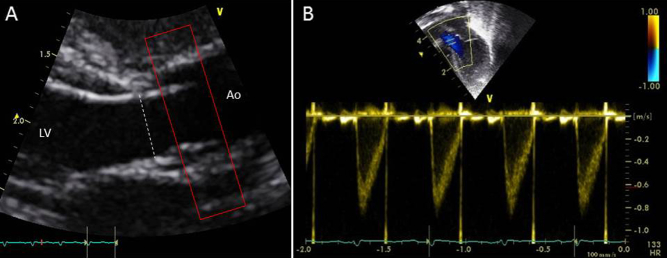
Echocardiographic quantification of LVO, with **(A)** diameter measured from the parasternal long-axis view and **(B)** velocity assessed from the apical view. Approximate location of PC MRI acquisition plane is also demonstrated (*red box*). *Ao*, Aorta; *LV*, left ventricle.

Aortic dimension was assessed from the parasternal long-axis view, with high-definition zoom to the aortic valve, and diameter was assessed at the valve hinge points at end-systole (Figure 1A, *dashed line*). Aortic flow velocity was assessed by pulsed-wave Doppler from an optimized apical five-chamber view, with the pulsed-wave Doppler gate placed at the level of the aortic valve (Figure 1B). Care was taken to minimize angulation between the Doppler beam and flow direction in the left ventricular outflow tract.

### SVC Flow Volume

**Figure 2:**
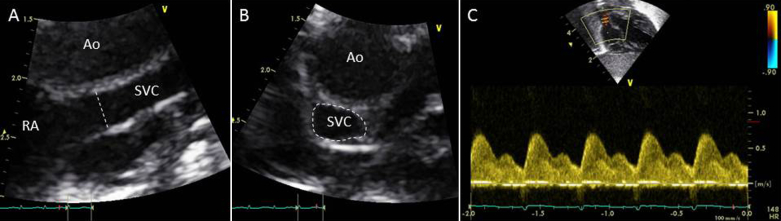
Echocardiographic quantification of superior vena caval flow, with **(A)** diameter measured from a modified parasternal long-axis view, **(B)** area measured from an axial view, and **(C)** velocity assessed from a low subcostal view. *Ao*, Aorta; *RA*, right atrium; *SVC*, superior vena cava.

SVC dimension was assessed using two distinct techniques. First, diameter was assessed from a modified parasternal long-axis view as initial described by Kluckow and Evans,^12^ hereafter described as the “sagittal” approach. High-definition zoom was used to focus on the superior vena cava as it begins to open up into the right atrium (Figure 2A, *dashed line*), with maximum and minimum diameters through the cardiac cycle taken from B-mode images. Because of concerns about the irregular shape of the Superior vena cava, we also measured vessel area directly from an axial view, again using high-definition zoom and tracing maximum and minimum cross-sectional area from the B-mode images (Figure 2B, *dashed line*). SVC flow velocity was assessed using pulsed-wave Doppler from a low subcostal view as described by Kluckow and Evans,^12^ with the ultrasound probe moved caudally until a clear length of the superior vena cava could be seen entering the right atrium, where the pulsed-wave Doppler gate was placed (Figure 2C).

In all cases, three to five consecutive cycles were analyzed, except in the case of SVC flow velocity, for which eight to 10 cycles were used to reduce the impact of respiratory variability. Angle correction of flow velocity was not used. All flow quantification was performed offline using EchoPAC software (GE Healthcare, Milwaukee, WI) by investigators blind to the PC MRI results.

### PC MRI Acquisition

PC MRI was performed using a 3-T scanner (Philips Medical Systems, Best, The Netherlands) using a specialized eight-channel pediatric body receive coil for infants weighing >2 kg or a small-extremity receive coil for infants weighing <2 kg. The methodology has previously been described,^8^, ^11^ but in summary, infants were allowed to fall into a natural sleep after a feeding, without the use of sedation or anesthesia. They had continuous monitoring of heart rate, oxygen saturation, and temperature. They received nasal continuous positive airway pressure or low-flow oxygen support as clinically indicated, and a specially trained pediatrician was in attendance at all times.

Single-slice PC MRI acquisition sequences with in-plane spatial resolution of 0.6 mm, slice thickness of 4 mm, repetition time of 5.9 ms, echo time of 3.1 ms, and 20 phases per cardiac cycle were used. Field of view and matrix were altered to maintain spatial resolution at 0.6 mm while minimizing scan duration. The velocity encoding was calibrated for the range of ±120 cm/sec for LVO sequences and ±60 cm/sec for SVC sequences. Three signal averages were used, allowing compensation of respiratory effects on cardiac output. Depending on heart rate and heart rate stability, the acquisition time for each two-dimensional PC MRI scan ranged between 2 and 4 min. No gating techniques were used to compensate for respiratory or other causes of motion.

**Figure 3:**
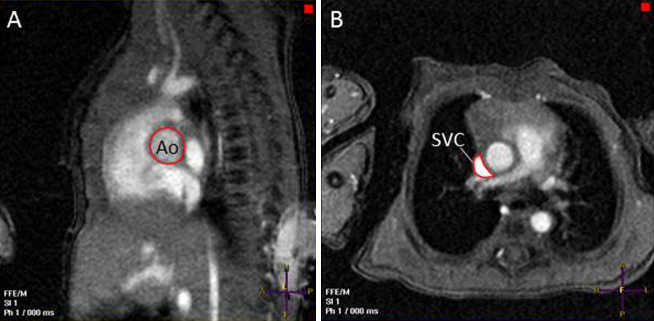
PC MRI quantification of **(A)** LVO at the level of the aortic valve and **(B)** superior vena caval flow at the level of the pulmonary trunk. *Ao*, Aorta; *SVC*, superior vena cava.

Pilot scans were acquired to view the vessels of interest to ascertain the straightest section of the vessel adequate for the slice thickness of the PC MRI sequences and to position the imaging plane perpendicular to the centerline of the vessel to minimize partial volume effects. LVO was quantified immediately distal to the level of the aortic valve (Figure 3A). Volume of flow in the superior vena cava was quantified at the level of the pulmonary trunk as the superior vena cava begins to open into the right atrium (Figure 3B).

Sequence analysis and flow volume quantification for PC data sets were performed using a commercial workstation (ViewForum; Philips Medical Systems). Automated vessel edge detection was used for all vessels of interest, with manual correction as necessary. Once defined in the first cardiac phase, the software tracks the vessel of interest over the cardiac cycle using edge detection algorithms. Flow is then calculated at each time point of the cardiac cycle, generating a flow curve and volume of flow value for each vessel of interest.

### Statistical Analysis

Measures of flow obtained by echocardiography and PC MRI were compared using simple linear regression and also as described by Bland and Altman^13^: the mean bias and limits of agreement (LOA, or “repeatability coefficient”; 1.96 × SD of differences) were calculated. Repeatability index (RI; LOA/mean of measures) was also calculated to allow comparison of repeatability between different measures.

## Results

Paired PC MRI and echocardiographic examinations were performed in 49 infants with a median gestational age of 32.57 weeks (range, 24.43–39.14) weeks and a median weight at birth of 1,750 g (range, 525–3,760 g). The median postnatal age at scan was 11 days (range, 1–84 days), with a median corrected gestational age of 34.43 weeks (range, 27.43–40 weeks) and a median weight at scan of 1,880 g (range, 660–3,760 g). The median interval between PC MRI and echocardiographic scanning was 3.67 hours (range, 0.25–33.0 hours), with PC MRI being performed before echocardiography in 46 of 49 infants. No infant had a significant change in clinical condition between PC MRI and echocardiography, and no infant received intervention for a blood transfusion or change in vasoactive medications or treatment for patent ductus arteriosus between the two scans. One infant was found to have a small (3-mm) muscular ventricular septal defect, and eight infants were found to have patent ductus arteriosus. Neither condition was considered to interfere with flow volume validation.

### Echocardiographic Assessment of LVO

**Figure 4:**
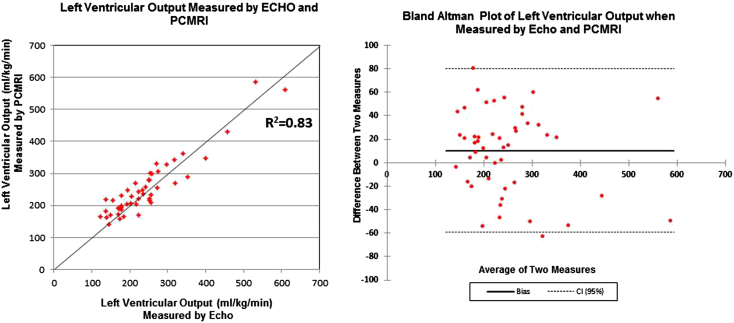
Comparison of LVO quantification by echocardiography and PC MRI in 48 newborn infants. *CI*, Confidence interval.

The means and standard deviations of aortic valve diameter, left ventricular outflow velocity-time integral (VTI; equivalent to stroke distance), heart rate, and LVO assessed using echocardiography and PC MRI are shown in Table 1. Significant disparities are seen in the echocardiographic and PC MRI measurements of vessel diameter and VTI because the two techniques quantify flow at slightly different sites in the left ventricular outflow tract. However, despite the disparities in the assessment of diameter and VTI, echocardiographic assessment of flow volume (LVO) showed a strong correlation with that by PC MRI (*R* = 0.91, *R*^2^ = 0.83; Figure 4). Bland-Altman analysis demonstrated a small mean bias between PC MRI and echocardiography of −9.6 mL/kg/min, with LOA of −79.2 to +60.0 mL/kg/min, corresponding to an RI of 28.2% (Figure 4).

**Table 1:** Mean ± SD of aortic diameter, left ventricular outflow VTI, heart rate, and LVO assessed by echocardiography and PC MRI in 47 newborn infants

Parameter	Aortic diameter (mm)	Left ventricular stroke distance (cm)	Heart rate for LVO assessment (beats/min)	LVO (mL/kg/min)
Echocardiography	6.0 ± 0.8	10.3 ± 2.0	143.8 ± 18.7	242.1 ± 99.3
PC MRI	8.7 ± 1.3	5.6 ± 2.3	141.0 ± 17.4	251.8 ± 92.2

### Assessment of SVC Flow with Diameter Measured in the Sagittal Plane

The means and standard deviations of SVC diameter (measured sagittally using echocardiography and axially using PC MRI), SVC VTI (stroke distance), heart rate, and SVC flow volume assessed using echocardiography and PC MRI are shown in Table 2.

**Table 2:** Mean ± SD of SVC diameter, SVC VTI, heart rate, and SVC flow assessed by echocardiography and PC MRI in 23 newborn infants

Parameter	SVC diameter (mm)	SVC stroke distance (cm)	Heart rate for SVC assessment (beats/min)	SVC flow (mL/kg/min)
Echocardiography	3.9 ± 0.9	14.5 ± 3.1	153.5 ± 17.7	118.4 ± 42.5
PC MRI	4.8 ± 0.8	7.3 ± 1.7	140.2 ± 19.1	101.8 ± 23.8

**Figure 5:**
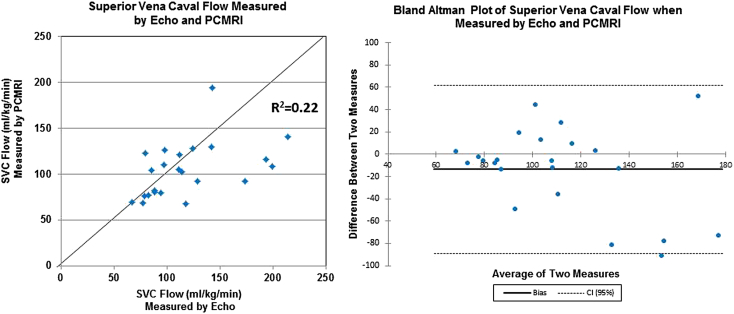
Comparison of SVC flow quantification by echocardiography (sagittal view) and PC MRI in 23 newborn infants. *CI*, Confidence interval.

Again, significant disparities were seen in the echocardiographic and PC MRI measurements of vessel diameter and VTI. However, echocardiographic assessment of volume of SVC flow showed a poor correlation with that by PC MRI (*R* = 0.47, *R*^2^ = 0.22; Figure 5). Bland-Altman analysis demonstrated a mean bias between PC MRI and echocardiography of −13.7 mL/kg/min (LOA, −89.1 to +61.7 mL/kg/min; RI, 68.0%; Figure 5).

Echocardiographic assessments of SVC dimensions (from a sagittal diameter measurement) systematically underestimated true SVC area: the mean PC MRI estimate of area was 18.9 mm^2^, the mean echocardiographic estimate of area 9.97 mm^2^, and the mean bias −8.9 mm^2^ (LOA, −19.8 to +2.6 mm^2^; RI, 77.4%). Echocardiographic assessments of SVC stroke distance systematically overestimated true SVC stroke distance. The mean PC MRI estimate of stroke distance was 7.28 cm, the mean echocardiographic estimate 14.5 cm, and the mean bias 7.2 cm (LOA, −2.0 to +12.4 cm; RI, 48.0%).

### Assessment of SVC Area from an Axial View

Echocardiographic assessments of SVC area directly from the axial view (mean, 16.7 mm^2^) more closely reflected SVC area as assessed by PC MRI (mean, 18.4 mm^2^). Calculating SVC flow volume from axial area measurement and applying a 50% reduction to stroke distance to compensate for systematic overestimation of VTI by echocardiography gave a slightly improved correlation with PC MRI (*R* = 0.54, *R*^2^ = 0.29). Bland-Altman analysis demonstrated a mean bias between PC MRI and echocardiography of 2.6 mL/kg/min (LOA, −53.4 to +58.6 mL/kg/min; RI, 54.5%).

### Variability in Heart Rate

Infants' heart rates tended to be slightly higher during echocardiography than PC MRI (147 vs 141 beats/min, *P* = .054) and showed significant variability (*r* = 0.69; LOA, −34.7 to +22.5 beats/min; RI, 19.9%), potentially reflecting differing levels of arousal.

### Scan-Rescan Repeatability of Quantification of SVC Flow by Echocardiography

Scan-rescan repeatability of echocardiographic quantification of volume of SVC flow was assessed in an additional cohort of 20 infants with a median corrected gestational age of 31.84 weeks (range, 26.71–36.57 weeks) and a median weight at scan of 1,518 g (range, 600–2,800 g). The mean VTI was 11.8 ± 3.6 cm, with mean bias on repeat scanning of 0.19 cm (LOA, −3.9 to +4.3 cm; RI, 34%). The mean SVC diameter measured sagittally was 3.9 ± 1.2 mm, with mean bias on repeat scanning of 0.01 mm (LOA, −0.49 to +0.47 mm; RI, 12%). Squaring of diameter to estimate area from sagittal SVC measurements showed mean bias of 0.0 mm^2^ (LOA, −4.0 to +4.0 mm^2^; RI, 29%). The mean SVC area measured axially was 16.1 ± 6.7 mm^2^, with mean bias on repeat scanning of −0.3 mm^2^ (LOA, −2.7 to +2.0 mm^2^; RI, 14%).

## Discussion

Improvements in circulatory care in preterm newborns are urgently required,^14^, ^15^ but progress is limited by inaccuracy in regularly used circulatory biomarkers. There is increasing acceptance that arterial blood pressure is a poor surrogate of circulatory health during the circulatory transition of preterm infants. Arterial blood pressure shows little if any association with the volume of systemic blood flow^16^ and has an uncertain relationship with the volume of cerebral blood flow,^17^, ^18^ and trials of randomized interventions on the basis of blood pressure thresholds have shown no apparent benefit on long-term outcomes.^19^, ^20^

Echocardiographic techniques in newborns can be performed in real time at the bedside, by a range of appropriately trained personnel, and if performed carefully need not significantly disturb infants' cardiorespiratory status.^21^ Although there is significant variation in the use of targeted neonatal echocardiography at different centers,^4^ there is an increasing appreciation that the techniques may be able to advance care.^3^, ^5^, ^22^, ^23^, ^24^, ^25^, ^26^

We have presented the first PC MRI validation of multiple echocardiographic measures of blood flow volume in preterm infants. Although PC MRI is not a flawless gold standard, it produces accurate measures of flow volume that can be validated ex vivo^8^ and that show repeatability in the neonatal population far superior to that seen with echocardiography.^27^ LVO and SVC flow volume as assessed by PC MRI had scan-rescan RIs (equivalent to the 95% confidence interval) of 11.5% and 12.8%, respectively.^11^ In the absence of any true gold standard, and acknowledging that all other “gold standards” such as the Fick method and thermodilution, also have intrinsic variability,^28^, ^29^ we feel that PC MRI is the best comparator currently available for the validation of echocardiographic findings in this population.

Our findings broadly support the use of echocardiographic assessments of LVO, which appear to be performed with a relatively high degree of accuracy. Although there was a significant disparity between echocardiography and PC MRI measures of aortic diameter and VTI, it should be noted that echocardiography quantifies flow at a single point at the aortic valve annulus, the narrowest part of the outflow tract, whereas PC MRI quantifies flow distal to this point at which the aortic sinuses increase vessel diameter (Figure 1) and for which stroke distance averaged over the entire vessel will necessarily be lower. When absolute volume of LVO was calculated from the respective echocardiographic and PC MRI parameters, there was a much closer association, with an *R*^2^ value of 0.83. The statistical correlation between echocardiographic and PC MRI measures of LVO might be somewhat enhanced by the wide range of LVO values seen in the newborn population, in which a patent ductus arteriosus frequently leads to supraphysiologic levels of LVO.

Assessment of repeatability by the method suggested by Bland and Altman^13^ is not affected by the range of values within a population and provides a more robust assessment of a test's performance. Echocardiographic assessment of LVO in the neonatal population showed a tendency to underestimate flow (mean bias, 9.6 mL/kg/min), but this was a disparity of <4% of the flow volume. Although LOA of about 70 mL/kg/min (approximately 28% of flow) are higher than desirable, it should be appreciated that echocardiography and MRI were performed up to 33 hours apart, and a degree of variability may be due to true biologic fluctuation rather than inconsistencies in measurement. To highlight this, we note that the LOA for heart rate, a variable presumably measured with complete accuracy, were 29 beats/min (an RI of 19.9%) in this cohort. The small size of infants in this cohort (mean weight at scan, 1,890 g) will also mean that minor alterations in transducer position are likely to produce significant errors.^5^ However the validation against PC MRI for LVO seen in this cohort (*r* = 0.91) actually compares well with PC MRI validation of two-dimensional Doppler assessment of left ventricular stroke volume in adults (*r* = 0.80),^30^ three-dimensional Doppler assessment of mitral valve flow in children (*r* = 0.92),^31^ and even three-dimensional Doppler validation of left ventricular stroke volume in anaesthetized animals (*r* = 0.91).^32^

Echocardiographic assessments of SVC flow volume appear to be less robust and consequently should be used with caution in the clinical environment. Echocardiography systematically underestimated the area of the superior vena cava, with area measures being about half those demonstrated by PC MRI. Because the true cross-sectional area of the superior vena cava would not be expected to change significantly in the area visualized, we feel that errors in SVC dimension quantification are partly related to the asymmetric nature of the vessel. For much of its course, the superior vena cava is closely adherent to the ascending aorta, and it molds to the shape of this higher pressure vessel. Cross-sectional images of the vessel visualized by echocardiography and PC MRI are shown in Figures 2B and 3B. Imaging in a sagittal plane and using an imaging window as close as possible to the midline as originally described^12^ will lead to estimation of SVC diameter toward the narrower “tail” end of its often crescent-shaped cross-section (Figure 3B). Although it is possible that others may be able to perform the image acquisitions from the sagittal view with improved repeatability compared with our group, it should be noted that there is significant variation in normative values produced from different centers^12^, ^33^ and that even assessment of SVC flow volume from a single prerecorded image acquisition has been shown to have significant variability.^34^

Echocardiography also systematically overestimated SVC stroke distance, with stroke distance measures being about double those demonstrated by PC MRI. These errors presumably represent nonlaminar flow in the vessel. This can best be demonstrated in the pulsed-wave Doppler images, in which rather than seeing an “open envelope” of flow (as seen in the aorta; Figure 1B), there is a “filled envelope” of flow (Figure 2C) in the superior vena cava, showing that not all flow in the vessel is accelerating to near the maximal velocity. Because stroke distance is calculated from the area under the velocity-time trace, all flow will be assumed to be at the maximal velocity.

By combining measures of dimension (which is underestimated by echocardiography) and VTI (which is overestimated by echocardiography) to produce estimates of SVC flow, the resultant flow measures are in a biologically plausible range but with a high degree of variability.

Direct assessment of SVC area from the axial view was more accurate and showed improved scan-rescan repeatability compared with measures of SVC area produced by squaring diameter measures taken from the sagittal view (RI, 14% vs 29%). However, even using the improved axial measurement of SVC flow and a corrected measure of stroke distance, the 95% confidence limits for the echocardiographic estimation of SVC flow volume were still 54.5% of the PC MRI measure. This may be related to errors in the assessment of VTI from the subcostal view (scan-rescan RI, 34%).

## Conclusions

Although echocardiographic assessment of LVO appears to be relatively robust, it is of limited clinical value in the neonatal unit setting, in which the majority of sick preterm infants will have patent ductus arteriosus, meaning that LVO does not represent systemic blood flow and is in fact a better marker of pulmonary flow volume. Although the volume of right ventricular output may be considered a marker of systemic blood flow, it also includes the volume of intra-atrial shunting, which may be as much as 50% of LVO in a premature newborn.^35^ Assessment of SVC flow is predictive of adverse outcomes in populations of preterm infants^36^ and has significant potential to be used as outcome measures in clinical trials,^37^, ^38^ but in our opinion, its limited accuracy gives it limited clinical utility in identifying circulatory failure in individual infants. However, estimating the vessel area directly from the axial view offers some improvement in performance, and there is scope for the technique to be further improved in the future, such as by estimation of VTI from the suprasternal instead of the subcostal view.^39^
